# Electromagnetic stirring in a microbioreactor with non‐conventional chamber morphology and implementation of multiplexed mixing

**DOI:** 10.1002/jctb.4762

**Published:** 2015-07-17

**Authors:** Christabel KL Tan, Matthew J Davies, Daniel K McCluskey, Ian R Munro, Mauryn C Nweke, Mark C Tracey, Nicolas Szita

**Affiliations:** ^1^ School of Engineering and TechnologyUniversity of HertfordshireUK; ^2^ Department of Biochemical EngineeringUniversity College LondonUK

**Keywords:** microbioreactor, mixing, multiplexing, electromagnetic actuation, microfluidics priming

## Abstract

**BACKGROUND:**

Microbioreactors have emerged as novel tools for early bioprocess development. Mixing lies at the heart of bioreactor operation (at all scales). The successful implementation of micro‐stirring methods is thus central to the further advancement of microbioreactor technology. The aim of this study was to develop a micro‐stirring method that aids robust microbioreactor operation and facilitates cost‐effective parallelization.

**RESULTS:**

A microbioreactor was developed with a novel micro‐stirring method involving the movement of a magnetic bead by sequenced activation of a ring of electromagnets. The micro‐stirring method offers flexibility in chamber designs, and mixing is demonstrated in cylindrical, diamond and triangular shaped reactor chambers. Mixing was analyzed for different electromagnet on/off sequences; mixing times of 4.5 s, 2.9 s, and 2.5 s were achieved for cylindrical, diamond and triangular shaped chambers, respectively. Ease of micro‐bubble free priming, a typical challenge of cylindrical shaped microbioreactor chambers, was obtained with a diamond‐shaped chamber. Consistent mixing behavior was observed between the constituent reactors in a duplex system.

**CONCLUSION:**

A novel stirring method using electromagnetic actuation offering rapid mixing and easy integration with microbioreactors was characterized. The design flexibility gained enables fabrication of chambers suitable for microfluidic operation, and a duplex demonstrator highlights potential for cost‐effective parallelization. Combined with a previously published cassette‐like fabrication of microbioreactors, these advances will facilitate the development of robust and parallelized microbioreactors. © 2015 The Authors. *Journal of Chemical Technology & Biotechnology* published by John Wiley & Sons Ltd on behalf of Society of Chemical Industry.

## INTRODUCTION

Microbioreactors, i.e. miniaturized bioreactors with sub‐milliliter reactor volumes, have emerged as a new tool for early bioprocess development.^1^, ^2^ They enable the rapid evaluation of multiple culture conditions while monitoring relevant fermentation variables*,* such as dissolved oxygen, pH, and optical density (OD; as an indirect measure of cell density),^3^, ^4^, ^5^ which are typically detected using optical means. The small volumes of microbioreactors also result in less media consumption and lower set‐up costs, in particular once the potential for multiplexing and parallelization has been realized.^3^, ^4^ When developing novel microbioreactor designs, it is therefore reasonable to consider the requirements for parallelization at an early stage of development.

Mixing lies at the heart of bioreactor operation – regardless of the size or scale of the reactor – and the development of novel mixing methods (and their successful implementation) is, therefore, central for the further advancement of microbioreactor technology. Mixing at the microscale is challenging due to the absence of turbulent flow conditions that are conducive to good mixing. And while many microfluidic mixing methods have been developed and characterized to overcome this drawback,^6^, ^7^, ^8^ most of these methods are not (or not directly) applicable to microbioreactors. ‘Passive’ microfluidic mixers (‘passive’ because there is no ‘active’ external energy input to the device other than the one to drive the fluid flow) typically operate at flow rates larger than the dilution rates useful for example for a continuous culture microbioreactor; and would not be applicable for batch operation mode. ‘Active’ microfluidic mixers induce disturbances using an external energy field, yet were still mostly designed to mix converging streams of fluid flow.

Mixing in submilliliter microbioreactors^1^ has been accomplished primarily either by shaking,^9^, ^10^, ^11^ stirring,^3^, ^12^, ^13^ or a peristaltic motion of one of the reactor chamber walls.^4^ All three have their own challenges for miniaturization: to avoid cross‐talk between neighboring wells (of the optically detected fermentation variables), for shaken microbioreactors (or shaken microtiter plates), the diameter of the shaking motion should be smaller than the diameter of a single well.^14^ Smaller microbioreactors have been realized using peristalsis^4^, ^5^, ^15^ or stirring; with stirrer bars in both pinned^3^ and free‐floating^16^ forms. Only a few of them have been multiplexed;^3^, ^4^ probably due to the expense and complexity of the actuation systems. Those microbioreactors in which multiplexing has been demonstrated required either separate motors^3^ or peristaltic pumping drivers^4^ for each microbioreactor, limiting scalability, and increasing the expense and the complexity of external pressure control manifolds for internal fluid control.

In this contribution, we investigate an electromagnetically actuated stirring method, which provides direct, external control over the direction and range of motion of an internal magnetic stirring bead. We demonstrate how this stirring method furthers the flexibility in the design of reactor chambers in addition to the flexibility already demonstrated using a ‘cassette‐like’ format.^17^ For this, we compare mixing in a cylindrically‐shaped reactor with unconventional reactor designs. Furthermore, we analyze the priming, or filling with liquid, of these microfluidic devices, which is typically a challenge for microfluidics,^18^, ^19^, ^20^ yet key for robust operation of these reactors. Finally, to underscore how electromagnetic actuation lends itself to further parallelization, we show a duplex demonstrator.

## MATERIALS AND METHODS

### Microbioreactor fabrication and assembly

All parts made from polycarbonate (PC) (RS Components, UK) were fabricated by micro‐milling (M3400E, Folken Industries, USA). Membranes (100 µm thick) were made from poly(dimethylsiloxane) (PDMS; Sylgard 184, Dow Corning, UK) by spin‐coating (P6708D, Specialty Coating Systems, USA) onto a silicon wafer (500 rpm, 20 s, 100 rpm s^−2^; 700 rpm, 30 s, 200 rpm s^−2^); the membrane frame PDMS structures were cast in a PC mold. All PDMS parts were cured in an oven (90 °C, 2 h). Milled parts were designed using a 3D CAD package (Solidworks, Dassault Systems, France) with CNC code being generated from the completed designs using a CAM program (Mastercam X4, Mastercam, USA). Thermal bonding of PC was performed at 135 °C (UFP400, Memmert). PDMS parts were bonded using air plasma‐assisted bonding (PDC‐002, Harrick Plasma, USA).

**Figure 1:**
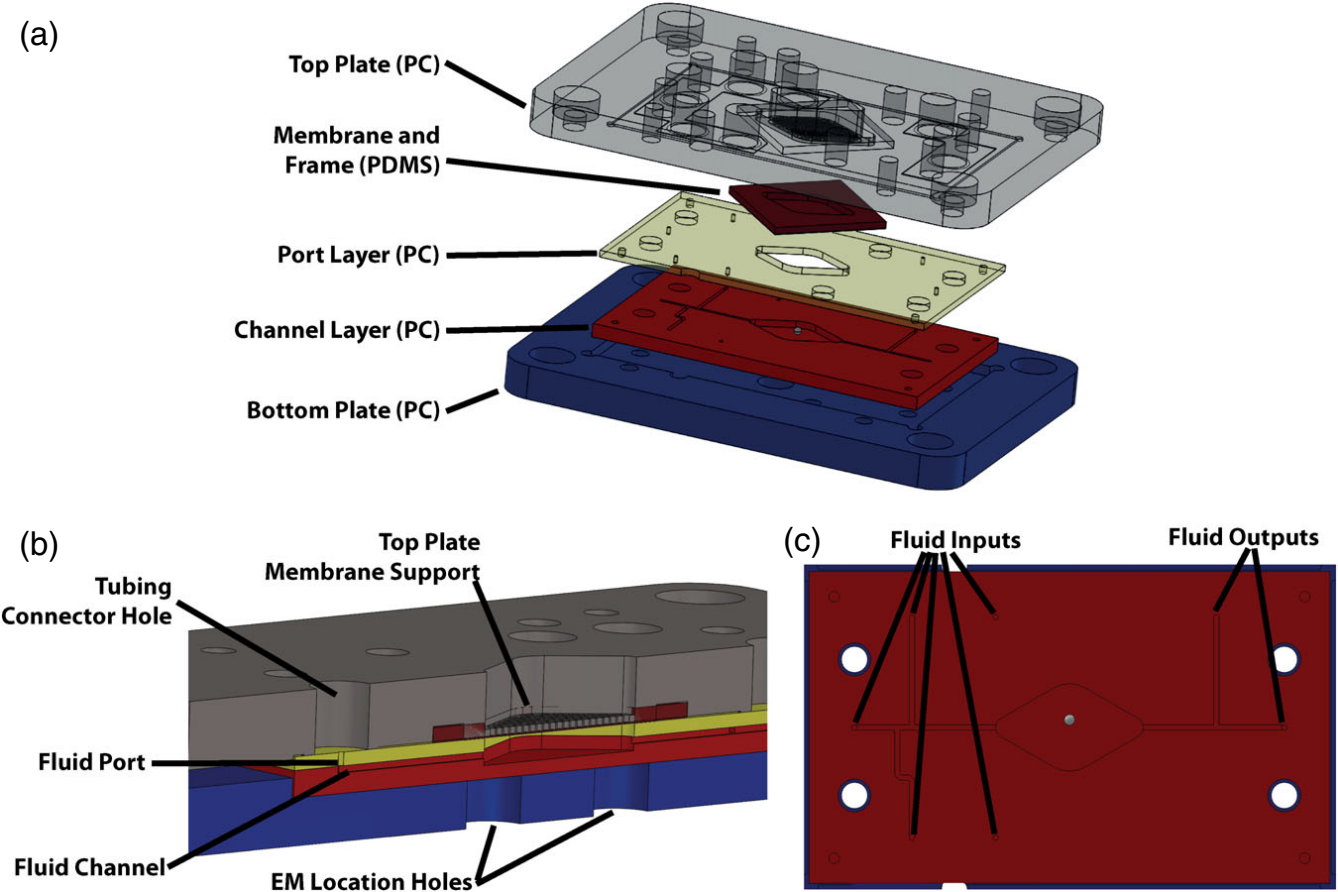
(a) Microbioreactor ‘cassette’ schematic; (b) cross‐section of the schematic highlighting key structural features; and (c) schematic of the microbioreactor fluid paths highlighting the five fluid inputs and two fluid outputs.

Each device was designed for assembly in a 'cassette'‐like format as detailed in Davies *et al*.^17^ Briefly, a 5 mm thick PC 'cassette' top and bottom plate enclosed a 3 mm PC microbioreactor body, containing the reactor chamber and fluid input and output channels. A precisely machined structure, ringing the top plate membrane support, compressed the PDMS membrane, by 50 µm, onto the reactor chamber to form a fluid seal (Fig. 1(a)). The microbioreactor body was manufactured by thermally bonding two PC layers, one for the channels (2 mm thick), and one for the fluidic ports (1 mm). Assembly of the device was completed by clamping the microbioreactor body and the PDMS membrane (Fig. 1(b)) by the cassette top and bottom plate with eight M3 screws tightened to a torque of 45 Ncm.

**Figure 2:**
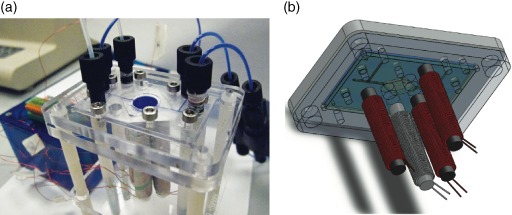
(a) Photograph of microbioreactor with electromagnets located below the reactor chamber, and (in background) the control electronics. (b) Schematic representation of positioning of electromagnets below the microbioreactor.

The cassette provided the interface with the external components for mixing, optical analysis, and fluid supply (Fig. 1(a)). For this, the cassette bottom plate had holes to accommodate the electromagnet cores (Fig. 1(b)), with hole spacing experimentally optimized for bead movement while providing for air flow to cool the electromagnets, and the optical fibre for measuring OD. The top plate enabled up to five input and two output tubing connectors (Upchurch Scientific, USA) to be attached (Fig. 1(c)). Figure 2 shows the assembled device and the positions of the electromagnets with regard to the assembled device.

**Figure 3:**
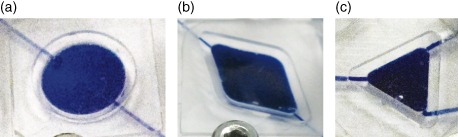
Microbioreactor chamber morphologies, for which electromagnetically actuated mixing was demonstrated, containing the 1 mm magnetic bead, and filled with water blue dye. (a) 157 µL volume circular chamber; (b) 146 µL volume diamond shaped chamber; (c) 71 µL volume triangular chamber.

Three distinct chamber designs were fabricated to evaluate the versatility of the electromagnetic mixing presented: a circular chamber with 10 mm diameter similar to Szita *et al*.^3^ (Fig. 3(a)); a diamond shaped chamber with 60° angles at inlet and outlet, with a volume similar to that of the circular chamber (16.1 mm long × 9.3 mm wide, Fig. 3(b)); and an equilateral triangular chamber with a volume approximately half that of the diamond chamber, but with similar side dimensions (9.3 mm long) to demonstrate complete mixing within sharp corners (Fig. 3(c)). All chambers were 2 mm deep, yielding chamber volumes of 157 µL, 146 µL, and 71 µL, respectively.

### Electromagnet fabrication and control

The electromagnet cores were formed from 5 mm diameter ‘silver steel’ rods (Roebuck, UK) conforming to BS1407. The electromagnet tips were ground to a 120° included angle cone with a slightly blunted point. They comprised 480 turns of 0.25 mm (conductor) diameter single‐core copper wire. Wire was wound in four layers over a length of 35 mm centrally located on a 45 mm long core. Stirring was implemented by moving a 1 mm diameter permanent magnet bead (Earthmag GmbH, Germany) between points directly above successively energized electromagnets. The bead was separated from the electromagnets by the 1 mm thick floor of the reactor chamber.

The electromagnet energization sequence for the single chamber mixer was achieved using an Arduino Mega2560 Microcontroller platform (www.arduino.cc). It provides the additional i/o lines required to address the driver ICs of the electromagnets. Code (scripts) was written in the Arduino derivation of the C++ based Wiring development environment. Electromagnet drive in both the single and dual chamber units was implemented by a H‐bridge motor driver (VNH2SP30, ST Microelectronics) incorporated into circuit board MD03A (Pololu, Las Vegas, USA). High speed, dual rectifier diodes (MBR20L100CT, Taiwan Semiconductor), incorporated at each output, constrained the outputs in the range between power and ground, in order to provide extra IC protection against back‐EMF (electromagnetic force) resulting from electromagnet switching.

### Experimental set‐up

The microbioreactor fluid system utilized to demonstrate mixing used three (of the five available) inputs and both outputs of the reactor. During mixing experiments, two of the inputs were connected to a single syringe pump (KDS200, KD Scientific, USA), supplying reverse osmosis purified (RO) water, with the third input connected to a separate syringe pump supplying dye. Plastic syringes (5 mL, Plastipak, Becton‐Dickinson, Fisher, USA) were connected to the microbioreactor via FEP tubing (554–2987, VWR International, UK) fluid system. Back‐pressure regulators (P‐790, Upchurch Scientific, USA) were connected to both outputs to prevent bubble formation.

### Analytical methodology

#### Mixing

Priming of the fluid system and all devices was performed at 50 µL min^−1^ using 70% ethanol, for efficient bubble removal via lower surface tension than aqueous solutions. Following priming with ethanol, the devices were flushed with RO water at 50 µL min^−1^. Set‐up for each measurement involved reducing the flow of water to 2 µL min^−1^ while starting the flow of methyl blue dye (M6900, Sigma Aldrich, UK) at 50 µL min^−1^ to ensure that the dye filled the chamber rapidly and to reduce diffusion time. Stirring was initiated once the dye had filled approximately 25% of the chamber, which was judged by reference to the liquid reaching consistent points along the walls of the chamber (see also ESI 1 for mixing without electromagnetic stirring).

**Figure 4:**
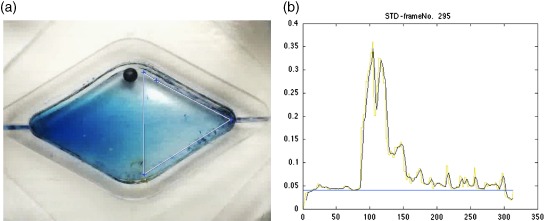
(a) Image from a video still, generated by the mixing analysis software, showing the user‐defined area of interest (faint triangle), in which the pixels were analyzed to determine the degree of mixing. (b) The resulting plot of the standard deviation of the red channel, within the area of interest, of all the frames from the video from which the still in Fig. 4(a) was taken with the frame number shown on the x‐axis.

Each mixing experiment was recorded with a USB microscope (VMS‐004D, Veho, UK). Two programs written in MATLAB (MathWorks Inc, USA) were then used to analyze the videos. The first converted the videos into a series of images in jpg format. The second program measured the value of a single color channel for every pixel within a user‐defined region (Fig. 4(a)) of the series of images, averaged those values, then plotted the raw and the normalized standard deviation of the pixel values (Fig. 4(b)). Mixing time was defined as the number of images between the start of mixing and the simple moving average (with a lag of five previous data points) of the normalized standard deviation reaching a stable value, with each image representing a period of 40 ms given a frame rate of 25 frames per second (fps).

#### Electromagnet Heating

Results from the electromagnet heating experiments were used to define the temperature setting for the incubator. Electromagnet heating experiments were performed with the bioreactor set‐up as per a fermentation, but with the fluid system filled with water. Prior to temperature measurements all system components were equilibrated to room temperature. Temperature measurements were made using a thermocouple, attached to a multimeter (Fluke 179, Fluke, USA), placed in contact with the cassette top plate and then with the electromagnets. Measurements were taken until the temperature stabilized at the cassette top plate. If the temperature surpassed 37 °C, the measurements were stopped; the incubator had only heating capability.

## RESULTS AND DISCUSSION

### Device designs

Three chamber designs were tested: circular, diamond and triangular. The circular chamber design provided a comparison with a similar chamber design that had previously been used by Szita *et al.,* 3 and for which mixing, using a pinned stirrer bar, had been quantified. Therefore, the diamond chamber design dimensions were chosen to maintain approximately the surface area to volume ratio of the circular chamber. The dimensions of the triangular chamber were chosen to enable closer examination of the figure‐8 actuation pattern on mixing in the diamond reactor; two triangular chambers placed back‐to‐back yield the same dimensions as the diamond chamber. With these dimensions, the loop pattern within the triangular chamber provides a comparison with ‘half of a figure of 8 pattern of the diamond chamber’. Maintaining the same surface area to volume ratio between the triangular and diamond chamber would not have enabled comparison of mixing, given the limited actuation patterns available in the triangular chamber.

### Liquid priming

**Figure 5:**
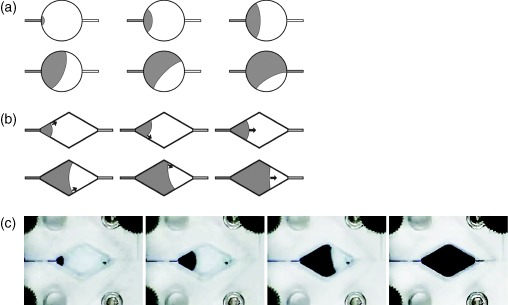
Comparison of the circular and diamond chambers during priming: (a) schematic of the meniscus movement as the circular chamber is primed, one edge of the meniscus becomes pinned, and a bubble trapped; (b) schematic demonstrating how the diamond chamber primes without trapping bubbles due to sequential advancement of the meniscus if one edge does become pinned; and (c) stills from a video showing the diamond chamber priming with no residual bubbles despite the meniscus being (temporarily) pinned to the top edge in the third image.

Velocity, inertia and the surface tension of the fluid, and the surface energy and wettability of the solid determine the movement of the fluid meniscus along the chamber.^21^ Given an ideal surface, the Weber number and Young's equation would be sufficient to determine the shape of the meniscus based on the relationship between the meniscus and the reactor surface. However, surface roughness and local changes in surface chemical properties introduce a hysteresis effect^22^ that results in the meniscus effectively being ‘pinned’, or held in place, by a surface irregularity and can lead to increase of contact angle of up to 50°.^23^ Surface roughness, resulting from device fabrication‐related hysteresis enables, when combined with the circular symmetry of the cylindrical chamber, the meniscus to rotate about the pinned edge. If this occurs, a complete fluid circuit from input to output may be made despite the presence of a residual bubble along one wall (Fig. 5(a)). However, within the diamond shaped chamber the contact angle of a meniscus pinned on one wall, as chamber filling progresses, will increase at the ‘pinned’ meniscus' edge until it reaches a critical angle. Unlike the cylindrical chamber, the diamond shaped reactor chamber does not possess circular symmetry and therefore as the trailing, ‘pinned’ (third panel of Fig. 5(b)), edge of the meniscus reaches the critical angle it will advance. The previously pinned edge initially advances more rapidly than the leading edge, having the side effect of making it less likely, by virtue of momentum, that the edge of the meniscus will be pinned again, until the meniscus edges contact angles are again similar. That is, the meniscus will advance towards the outlet with transient delays followed by jumps on one edge with the average motion of the meniscus being relatively even on both sides of the chamber. Therefore, priming the diamond chamber is much simpler than priming the cylindrical chamber.

The circular chamber required the aid of 70% ethanol and orientating the device vertically to allow bubbles to rise to the output channel for efficient priming. In contrast, aqueous bubble‐free priming of the diamond chamber was straightforward (Fig. 5(c)) provided the chamber was filled from dry. Given the expected progression of the fluid meniscus and the presence of fluid channels at all of the corners, the triangular chamber was neither expected to, nor experimentally observed to, demonstrate any issues with bubble‐free priming.

### Implementation of electromagnetic stirrer

#### Design

To establish the electromagnetic stirring concept, we chose simple rod‐core electromagnets due to the ease of fabrication and the relatively simple emitted magnetic field. High silicon content ‘electrical steel’ would in principle be ideal for the electromagnet core. Such steels display lower eddy current losses, hence lower core heating, at the cost of mildly reduced magnetic saturation levels. However, these steels are difficult to machine due to their brittleness and hardness. Accordingly, the electromagnet cores reported here were formed from 5 mm ‘silver steel’ rod.

For electromagnet positioning, initial consideration indicated that an electromagnet configuration orthogonal to the fluidic plane would be preferable. Accordingly, the electromagnets' ‘footprint’ within the fluid plane would be minimized thus facilitating both the positioning of optical fiber sensing and subsequent progression to tightly‐packed multiplex chamber‐arrays.

#### Electronics

The electromagnet driving, H‐bridge VNH2SP30 allow software selection of electromagnet current direction, a feature that is used to provide flux direction reversal upon successive energizations of each electromagnet so as to minimize the development of permanent core magnetization which was observed to occur over some minutes in early tests with a unipolar drive topology. Given the actuated bead's spherical geometry, its adaptation to the flux reversals is seamless.

Finite element method magnetics (FEMM) (see ESI 2) calculates electromagnet inductance to be 12.5 mH with a coil resistance of 3 ohms resulting in a resistance–inductance time constant of 4.2 ms which is approximately 5 times less than the electromagnet on‐time of 20 ms reported here and hence, in conjunction with rapid drive IC switching times, judged to be negligible.

### Analysis of mixing behavior

Electromagnet actuated, magnetic bead movement is governed by several factors such as: the strength of the magnetic field, the time over which the field is present (‘on’ time), and the time between magnets being actuated (‘delay’ time). In addition, the distance between the EMs, and the fluid's viscosity and momentum all influence bead motion. As a result of the complex interacting nature of these multiple factors, iterative optimization of EM control sequences and determination of the minimum current and voltage, rather than theory driven development, was the most effective method for determining the settings under which initial experiments could be performed.

All mixing experiments were conducted using water and a water‐soluble dye. As a result all experiments are therefore only representative of the mixing behavior of Newtonian fluids with viscosity close to that of water. While mycelium forming organisms, such as S. tendae, may lead to non‐Newtonian fluid behavior,^24^ even relatively high cell density cultures typically exhibit Newtonian behavior.^25^ Therefore, it was not deemed necessary to test the stirring with non‐Newtonian fluids.

Video analysis to determine the mixing time was performed using a modified version of the method used by Rodriguez et al. 26 to analyze mixing within shaken cylindrical bioreactors. Due to the differing geometry, optical effects resulting from the inability to illuminate the reactor chamber as effectively as Rodriguez et al. 26 were present. Mitigating the greater noise that resulted from the optical effects led to the simple moving average (SMA) of the normalized standard deviation $\left(\sigma \left(G_{i,j}^{*}\right)\right)$ of the red channel being used for analysis of the mixing time. Noise levels in plots of the SMA of the $\left(\sigma \left(G_{i,j}^{*}\right)\right)$ were still too high to determine the specific point at which 95% mixing was obtained as per Rodriguez et al. 27 Therefore, an automated method of determining the frame at which mixing had started and a semi‐automated method of calculating the point at which the SMA of the $\left(\sigma \left(G_{i,j}^{*}\right)\right)$ plateaued were utilized.

Preliminary experiments were conducted into the effect of varying the distance between electromagnets, and the applied current, on bead movement. Airflow around the coils was essential to remove heat resulting from ohmic heating and magnetic core eddy‐current losses. A minimum distance was also required within the space enclosed by the four electromagnets to allow for future placement of optical fibers to enable optical interrogation of the bioreactor chamber. Below magnetic saturation, the magnetic field strength is linearly proportional to the applied current. However, the basic relationship between the field strength and distance is complex^28^ and further complicated by the permanent magnetic bead's attraction to the unenergized electromagnet's core.

**Figure 6:**
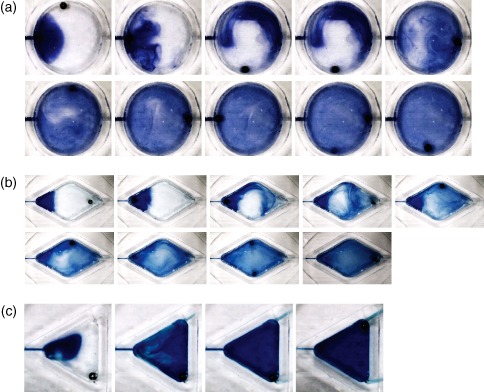
Stills taken from videos demonstrating mixing in (a) the circular chamber, (b) the diamond shaped chamber, and (c) the triangular chamber. The same electromagnet actuation pattern is used in both the circular and diamond shaped chamber, and the same on/off timing of the electromagnets is used for all reactor chambers. All stills were taken at 1 s intervals after the mixing sequence was started.

In both the diamond and circular reactor the magnetic bead was required to cross the center of the chamber (as opposed to following a loop around the wall (Fig. 6(a))) to ensure that mixing was complete; the loop pattern was observed not to mix fluid in the center of the chamber. The electromagnets were therefore arranged to form a square, with the sides of the square rotated by 45° with respect to the reactor body walls, and with each electromagnet positioned 6 mm from the center of the square. This resulted in the EMs being positioned approximately at the corners of the diamond reactor chamber; however, they are slightly offset due to the diamond reactor not being square. The chosen electromagnet configuration required a current of 0.3 A and a voltage of 8 V, in order for the magnetic field produced by each electromagnet to be strong enough to attract the magnetic bead.

The characterization of the performance of the mixer by the mixing energy (in addition to mixing time) is complex. Current, voltage and actuation time enable calculation of an average power input to the EMs (per cycle of the magnetic bead), yet the power is consumed by both the formation of the magnetic field and the electrical resistance of the windings. However, even the strength of the magnetic field is not directly related to the kinetic energy of the magnetic bead due to the complexity of the magnetic and physical interactions. Therefore, calculating the power input to the EMs cannot be extended to the power imparted by the motion of the magnetic bead to the system. Characterization of the mixing via measurement or modeling of the bead movement is further complicated by the complex nature of the actuation required to reduce the heat produced by the EMs.

The electrical power consumed by the electromagnet winding is dissipated as heat, known as joule or ohmic heating. Conduction of the heat, from the electromagnets, potentially raises the temperature of the reactor chamber, affecting the bacterial growth rate and the rate of evaporation. Therefore, measurement of the temperature of the reactor chamber, with differing duty cycles of the electromagnets, over extended periods of time, is necessary to enable accommodation of any temperature increase in order to perform a fermentation. While achieving a short mixing time was the primary aim, ensuring that a stable reactor temperature of 37 °C was achievable was vital.

**Figure 7:**
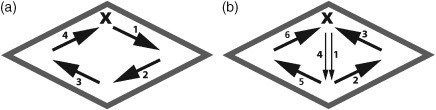
Schematic of the electromagnet actuation sequences: (a) loop; and (b) ‘figure of eight’. The electromagnets are placed at the corners of the diamond chamber and **X** indicates the starting point, i.e. the first electromagnet to be actuated of each sequence.

Following the optimization of the electromagnet configuration for both the circular and diamond shaped reactors, an Arduino script providing control over the actuation timing and sequence of the electromagnets was developed. A combined dual loop (Fig. 7(a)) and single 'figure of eight' (Fig. 7(b)) pattern, resulted from observations (not shown) that the fastest mixing, independent of EM timing, occurred when the on and delay times combined were below approximately 500 ms. This actuation pattern produced a 'vortex', by actuating the electromagnets with a loop pattern, then disrupted it by pulling the bead across the center of the chamber and briefly in the reverse loop direction, as occurs during the ‘figure of eight’ pattern. It was also observed while varying the timing sequence in the 'figure of eight' pattern that the further apart consecutive electromagnets were the longer the total on and delay timing sequence needed to be, with the on time increasing from 20 ms, for successive electromagnets around the chamber wall (approximately 8.5 mm apart), to 40 ms when the bead crosses the center of the chamber (electromagnets are 12 mm apart). Providing for separate pattern timing enables a faster timing sequence to be used during the loop pattern, producing a fast liquid rotation when successive electromagnets are closer together, than during the 'figure of eight' pattern, when the axial electromagnets are further apart.

The optimized EM electrical and spatial parameters and Arduino script were applied to the diamond chamber after being optimized in the circular chamber. As the triangular chamber does not provide for the pattern complexity that the diamond and circular chambers do, due to only three of the four EMs being used, a simple loop pattern was used. The electromagnet‐to‐electromagnet distance was maintained at 8.5 mm. Two different configurations were tested for the triangular chamber: electromagnets mounted at the corners; or mounted centrally along each side. Mixing was poor for the latter arrangement. An experiment was conducted in which the on and delay timing were the same, for both the loop and 'figure of eight' patterns, to enable comparison of the resulting mixing time within the circular (Fig. 6(a)), diamond (Fig. 6(b)), and the triangular chamber (Fig. 6(c)). The times used (on time = 100 ms, delay time = 50 ms) were deliberately chosen to produce slower mixing in order to provide greater contrast between the effectiveness of mixing in each chamber. Each image contained within each figure was taken with a 1 s gap. The circular chamber and diamond chamber demonstrate surprising consistency in the development of the fluid patterns produced and the total mixing time (9.6 s and 9.7 s, respectively), while the triangular chamber, despite using a loop electromagnet actuation pattern demonstrates much faster mixing (5.4 s). The smaller volume of the triangular chamber is likely to be responsible for this effect despite the presence of acutely angled corners in which mixing is difficult for the majority of alternative mixing methods. It was felt unnecessary to test a triangular chamber of similar volume to the diamond and circular chambers; the mixing times would have been significantly longer due to the aforementioned limitation in using only a loop actuation pattern with the resulting relatively stable liquid volume in the center of the chamber.

While the uniform timings stated above were useful for comparing mixing times between the three chambers, faster mixing was possible by reducing and varying the on and delay times within and between the loop pattern and the ‘figure of eight’ pattern. Various actuation times and repeats of each actuation sequence were tested and the mixing times measured (Table 1). As shown in Table 1, the fastest mixing times achieved in the circular and the diamond chambers were approximately 4.5 s and 2.9 s, respectively. While not as rapid in the circular chamber as that achieved using a free spin bar,^17^ further optimization of the electromagnet actuation in conjunction with computational fluid dynamics (CFD) modeling is anticipated to enable reduction in the electromagnetically actuated stirring mixing time.

**Table 1:** Electromagnet actuation patterns, on/off timing, number of loops and ‘figure of eight’ configurations, and the resulting average mixing times (n = 3) and corresponding standard deviations for three different chamber designs

Chamber design	Loop on (ms)	Loop delay (ms)	Fig 8 on (ms)	Fig 8 delay (ms)	N^o^ of loops	N^o^ fig 8's	Mixing time (s)	Standard deviation (s)
Circular	100	‐	‐	‐	‐	‐	4.75	1.6
Circular	‐	‐	100	‐	‐	‐	4.49	2.5
Circular	‐	‐	250	‐	‐	‐	8.33	0.7
Circular	100	100	100	100	4	1	5.99	1.3
Circular	100	50	100	50	4	1	5.57	0.1
Circular	100	150	150	150	2	1	11.72	0.9
Circular	100	50	100	50	2	1	7.47	1.8
Circular	50	10	100	50	2	1	4.54	0.3
Diamond	50	100	50	100	2	1	4.24	0.1
Diamond	50	50	50	50	2	1	4.17	0.6
Diamond	20	40	50	50	2	1	3.53	0.4
Diamond	20	40	30	70	2	1	2.87	0.2
Diamond	20	40	20	80	2	1	3.60	0.4
Diamond	50	10	100	50	2	1	3.20	0.2
Triangular	100	50	‐	‐	‐	‐	2.49	0.02

### Duplex mixing

**Figure 8:**
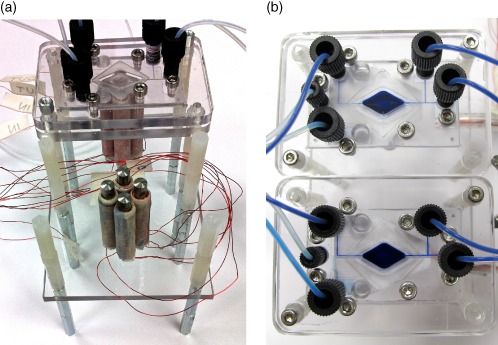
Duplex mixer set‐up with diamond shaped bioreactor chamber: (a) partially assembled showing the electromagnets in position for placement of the bioreactor; and (b) fully assembled with dye filling the chamber.

**Figure 9:**
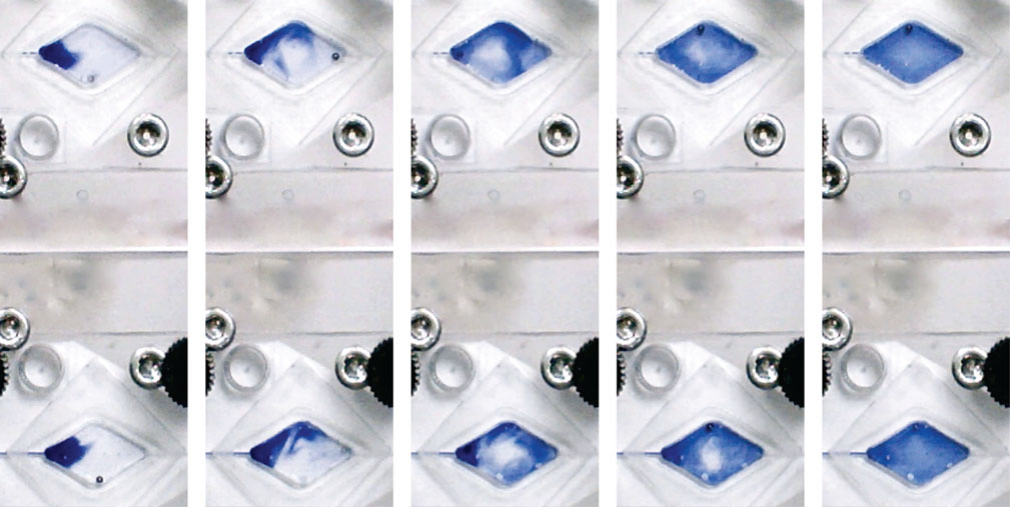
Video stills of mixing in the duplex diamond‐shaped chambers demonstrating similar mixing patterns between the chambers using a ‘figure of eight’ and loop mixing pattern.

As proof of concept for parallelization, a duplex microbioreactor set‐up was tested in which two separate diamond chamber microbioreactors with separate fluid supplies and electromagnet actuators were placed adjacent to each other (Fig. 8) and mixing tested for each independently, but simultaneously using the same methodology as applied to the single microbioreactor. The electromagnets for each microbioreactor were controlled by a simple script, running on an Arduino Mega 2560 that provided duplicate signals to equivalent electromagnets under each microbioreactor. As a result the same mixing pattern was applied to both bioreactors. As observed in Fig. 9 similar fluid patterns were observed in both bioreactors and the injected dye was completely mixed in both bioreactors after approximately the same length of time. This demonstrated that the mixing was equally effective in both bioreactors with no significant increase in system complexity as a result of the independent mixing of each bioreactor. Extending this implies that there is great potential for multiplexing of the microbioreactors using electromagnet actuated mixing. Furthermore, while the same mixing pattern was defined in the Arduino script developed for the proof of principle experiment presented here, the electromagnet configuration utilized enables different mixing patterns to be applied to the adjacent microbioreactors. This will allow varying the agitation between individual reactors and to study their impact on fermentation outcome.

## CONCLUSION

We successfully demonstrated the implementation of a novel electromagnet actuated, microbioreactor stirring method. Using electromagnets to move an impeller within a microbioreactor of 1 mL was shown before.^13^ In our design, electromagnets were positioned orthogonally underneath the reactor chamber, as actuators of a magnetic bead within the chamber. This enabled stirring in three different chamber designs: circular, diamond, and triangular shaped chamber (chamber volumes of 157, 146 and 71 µL, respectively). The advantage of implementing a diamond‐shaped chamber design was demonstrated by completely filling the chamber without requiring any particular priming procedure.

The electromagnet actuated stirring method will enable the development of parallel microbioreactors conducive to the growth of bacterial cultures: a minimum mixing time of approximately 3 s in the diamond shaped chamber and 4.5 s in the circular chamber is comparable with other microbioreactors.^4^, ^5^, ^16^ Furthermore, a duplex microbioreactor system illustrates the potential of this novel stirring method towards simpler and less expensive multiplexing of microbioreactors.

We are currently integrating further monitoring capabilities, such as sensors for dissolved oxygen. These will enable determination of the effect of actuation speed on the dissolved oxygen tension (DOT), which – combined with the CFD – will more fully characterize the behavior of this stirring concept. This characterization together with the measurement of the OD will enable further understanding of microbioreactor operation and fermentation outcomes.

## Supporting information

AppendixS1. ESI 1: Mixing times without electromagnetic actuated stirring

AppendixS2. ESI 2: Finite Element Modeling of the Electro‐magnetic Actuation
