# Supplementary material for: Electromagnetic stirring in a microbioreactor with non‐conventional chamber morphology and implementation of multiplexed mixing
Source: J Chem Technol Biotechnol. 2015 Jul 17;90(10):1927–36. doi: 10.1002/jctb.4762 (PMC4973846; doi:10.1002/jctb.4762)
Supplement: Supplementary file 1 — AppendixS1. ESI 1: Mixing times without electromagnetic actuated stirring [file JCTB-90-1927-s001.docx]

## ESI 1: Mixing times without electromagnetic actuated stirring

For comparison with the active mixing method, two separate experiments were conducted to determine the mixing time when the EMs are not actuated. Experiment 1 used the same set-up as for the active mixing experiments, yet without the EMs being actuated. Instead of a video, still images were taken, one every minute (Figure 1). For experiment 2, the reactor chamber was filled with water and a dye was flowed into the reactor chamber at 50 µl/min, again without the EMs being actuated.


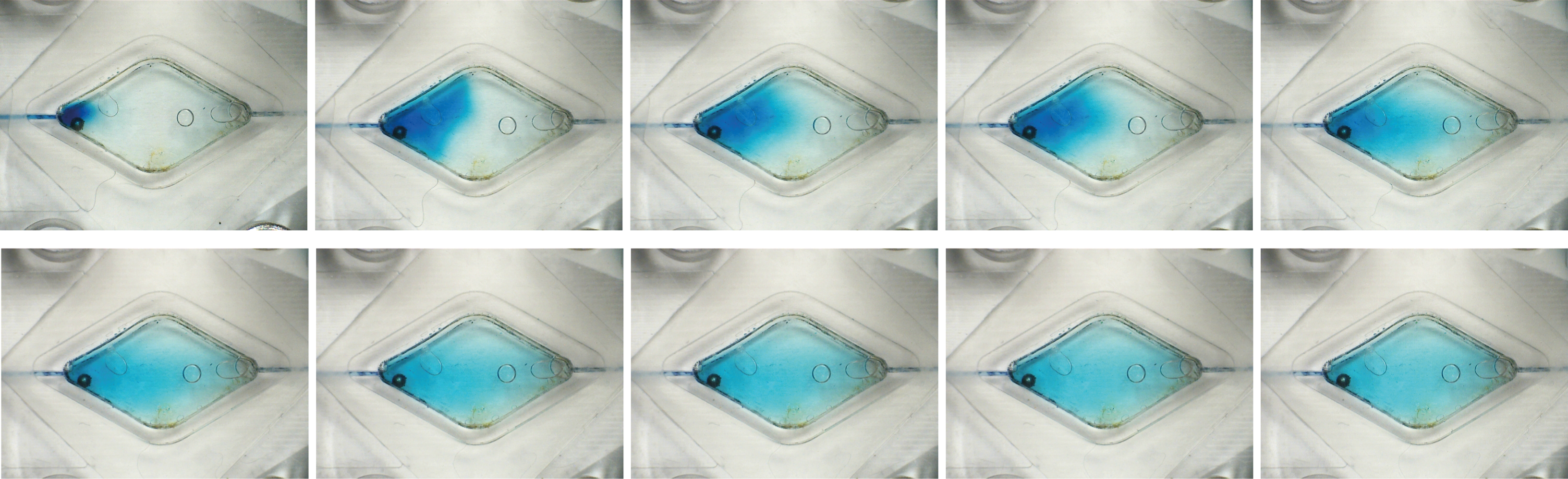


Figure 1. Sequence of images of diffusive mixing within a diamond microbioreactor chamber. The images run from left to right on the top row and continuing on the bottom row. The first image was taken at time 0 min, with subsequent images being taken every 10 minutes after that point.

Experiment 1 represents the scenario where mixing is solely dependent on diffusion, i.e. without convection from (external) fluid flow, and without active mixing. A mixing time of 93 minutes was measured (using the computational image analysis method detailed in the main manuscript). Experiment 2 provides an indication of the mixing without active mixing, but with net fluid flow through the chamber. For a flow rate of 50 µl/min, a mixing time of 18.7 seconds was measured.

These two experiments are also broadly representative of the two modes with which the presented micro bioreactor could be operated in principle: (i) batch cultivation (i.e. no fluid entering or leaving the reactor chamber), and (ii) continuous culture cultivation (i.e. continuous flow in and out of the reactor chamber, respectively).

For experiment 2, and to amplify the effect resulting from fluid flow, a very high flow rate of 50 µl/min was chosen; a continuous culture experiment would typically be performed at dilution rates below 0.5 h^-1^, resulting in flow rates less than 1.25 µl/min for a chamber volume of 150 µl. Despite the comparatively very high flow rate for experiment 2, the mixing time still significantly exceeded the time obtained when using electromagnetic actuation.

These experiments thus confirm that the EM-actuated stirring significantly reduces the mixing time, which is a critical parameter for suspension cultures.

Not only this, but due to optical artifacts it was possible to observe mixing occurring, but not possible to measure, the mixing well at the edge of the chamber, where diffusion is the limiting factor. This can be observed at the top and bottom of the chamber in the last image of ESI figure 1. As a result the mixing time, even at the rapid flow rates used in experiment 2, was actually longer than that measured here.
