# Supplementary material for: Electromagnetic stirring in a microbioreactor with non‐conventional chamber morphology and implementation of multiplexed mixing
Source: J Chem Technol Biotechnol. 2015 Jul 17;90(10):1927–36. doi: 10.1002/jctb.4762 (PMC4973846; doi:10.1002/jctb.4762)
Supplement: Supplementary file 2 — AppendixS2. ESI 2: Finite Element Modeling of the Electro‐magnetic Actuation [file JCTB-90-1927-s002.docx]

**ESI 2: Finite Element Modeling of the Electro-magnetic Actuation**

To inform on operation and electromagnet positioning, a public domain finite element method magnetics (FEMM version 4.2 – www.femm.info) model illustrating a single stirrer-bead step was constructed for a simplified two-electromagnet system^[[1]](#footnote-1)^. The plane of the 2D model is chosen such that the two electromagnets cores lie in it, as does the path of the moving bead. For convenience of modeling, the electromagnet to be actuated in the model was placed centrally, in an axis-symmetric configuration as shown in the cross-sectional drawing of Fig. 2. We also assume that the bead moves horizontally on the bed of the chamber, i.e. does not undergo vertical translation. This is a reasonable assumption given the small aspect ratio (depth over width) of the chamber, and a magnetic force that is either zero or attracts the bead towards the chamber bed.

The model does not simulate dynamic bead actuation; rather it is used to produce a series of static 'snapshots' of the field experienced by the bead at points through an electromagnetic bead actuation sequence.

The model is an axisymmetric problem with an Asymptotic Boundary Condition (an approximation to an unbounded solution). This boundary condition is applied in FEMM by selecting the *Mixed Boundary Condition* option requiring specification of the two parameters C_0_ and C_1_.

These are in this particular case:

$$C_{1}=0$$

$$C_{0}= \frac{1}{\mu_{r}\mu_{o}R}$$

where $\mu_{r}$ is the relative permeability of the region adjacent to the boundary, $\mu_{o}$ is the permeability of free space, and R is the radius of the modeled domain.

A generous domain radius of 100 mm was employed in addition to the *Asymptotic Boundary Condition*. A solver precision of 1 x 10^-8^ was employed. Meshing was separately specified for different regions in view of the local physical feature sizes and observed rate of change of field in initial tests. FEMM's meshing algorithm is either fully automatic or a maximum mesh element size can be specified. We chose to specify maximum mesh sizes. After a series of mesh-size sensitivity tests, a final maximum mesh size of 50 µm was used for the ball magnet, while that of the magnetic cores and remaining space was assigned a maximum mesh size of 250 µm. This resulted in a mesh of 31,927 nodes and 50,679 triangular elements with a mesh minimum angle of 30º. Denser meshes did not materially increase precision but increased demands on processor and memory. The second and adjacent passive electromagnet was modeled as a core alone as it was not necessary to energize it and hence the associated copper coil would not interact with the magnetic field differently from free-space. Core materials were chosen from the FEMM materials library as annealed carbon steel. A 1 mm neodymium-iron-boron (NdFeB) magnetic bead was incorporated resting on a plane 1 mm above the tips of the electromagnets. The bead material was chosen from the FEMM materials library as 37MGoe NdFeB. Electromagnet axes were spaced 8.5 mm apart, within a modeling plane positioned normal to the fluid flow plane, through an adjacent electromagnet-pair within the overall configuration of four electromagnets. These essential magnetic components are employed in the FEMM models to be discussed subsequently.


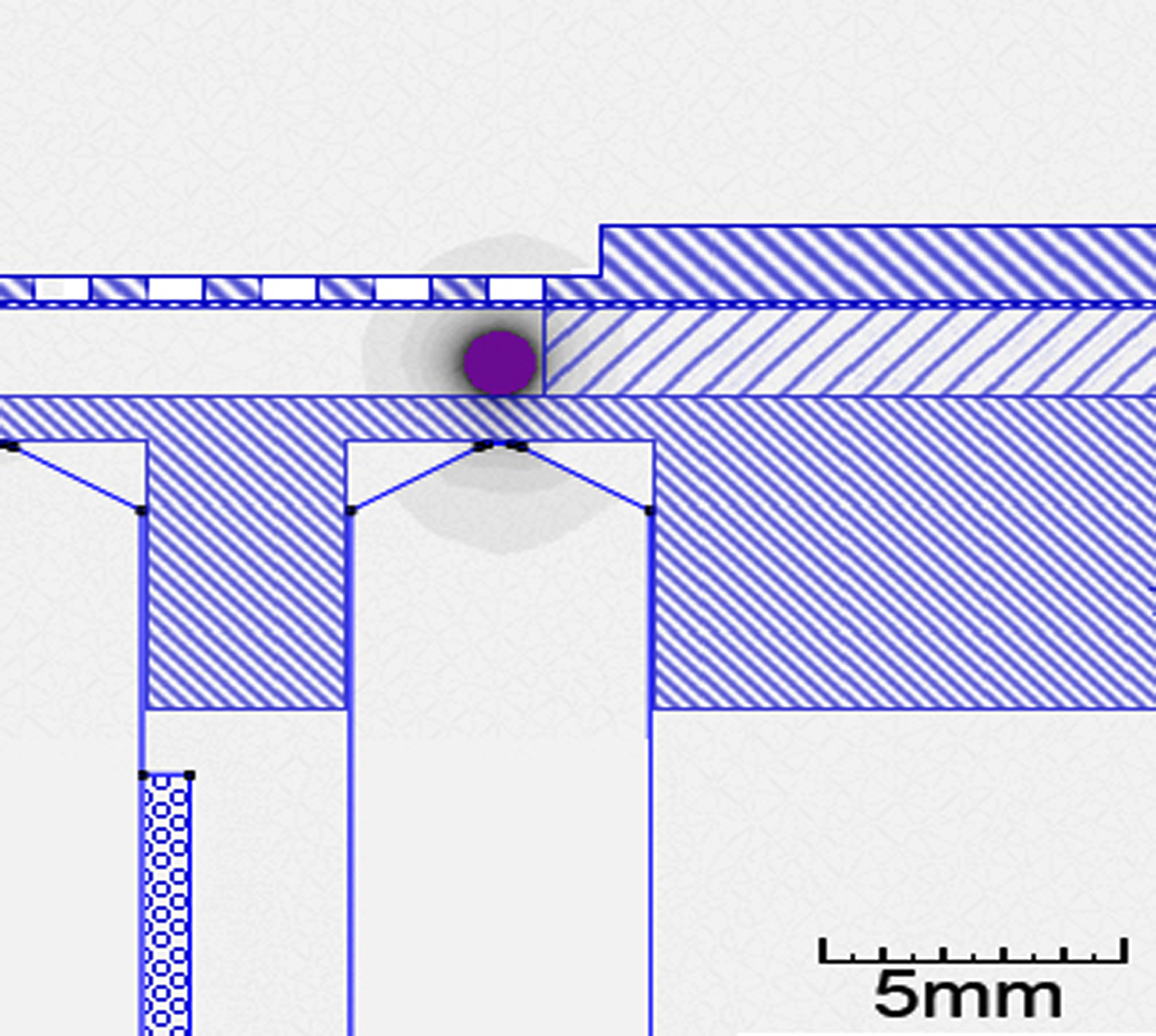


Figure 2. Simplified cross section of reactor structure showing the 1 mm magnetic bead at rest in the fluid chamber 1 mm above right hand electromagnet. The left hand electromagnet incorporates its coil structure, but this is omitted in the right hand magnet to agree with the magnetically modeled system. Shading illustrates the bead’s permanent magnetic field.

Fig. 3 (a-d) shows the magnetic field (B) experienced by the bead as it is attracted from the right electromagnet to the left one. Commencing with Fig. 3a), the bead starts at rest above the right electromagnet (having arrived there by a previous movement) which is de-energized, as is the left electromagnet. It can be seen the bead is immobilized by virtue of its own permanent magnetic attraction to the un-energized core. In Fig. 3b) the left electromagnet is energized and the bead is subjected to a composite B field resulting from both energized and un-energized electromagnets. Prima facie it would appear that the bead will remain located above the un-energized electromagnet due to greater flux density. However, as field lines display, the energized electromagnet provides a primarily horizontal vector component whilst the un-energized one contributes a primarily vertical one. Accordingly, as the bead is resting in point contact with a hard plane (the chamber floor) it will proceed to slide towards the energized electromagnet under the influence of the horizontal vector components. In reality, the ball will move through the liquid, thereby displacing it to cause stirring. A complexity of this process concerns the changing alignment of the bead’s magnetic axis to the composite field it encounters as it moves. This cannot be automated within the model, but is adjusted manually for each position. This alignment will effectively lock the bead rotationally at a given location and suggest that it will result in the bead sliding rather than rolling on the chamber floor. Whilst this was not the subject of current modeling, if this is the case, then in conjunction with a net downwards force (due to magnetism) it will result in frictional drag on the bead in addition to viscous drag during actuation. Both these forces will reduce bead velocity and hence potentially reduce ultimate mixing performance. However, as the bead moves towards the energized electromagnet this angle will change and this will contribute to forces at the point of contact between the bead and chamber floor resulting in some rotation. Fig. 3c) shows the bead being drawn strongly into the field of the energized electromagnet and Fig. 3d) shows the bead at rest in its new position prior to de-energization of the left electromagnet. Subsequent to de-energization, the left electromagnet will appear as does the right one in Fig. 3a).


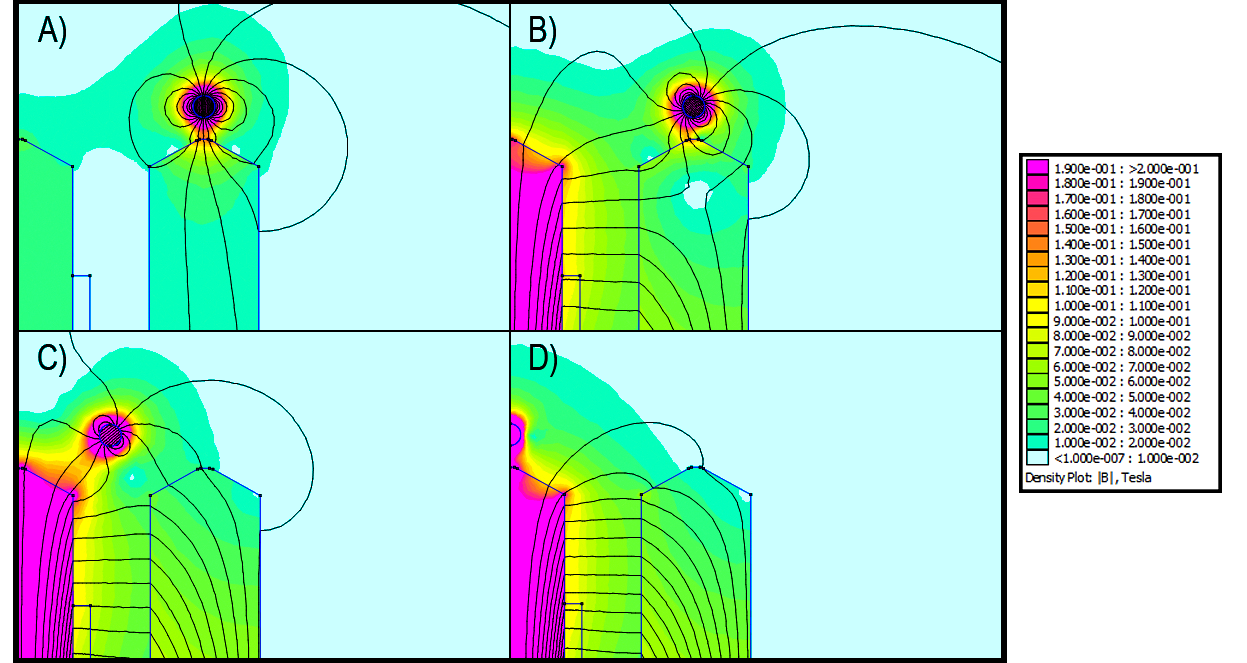


Figure 3.a). Bead attracted by permanent magnetism to un-energised electromagnet, b). left electromagnet energised, bead has yet to move, c). Bead moving into stong field of energised electromagnet, d). Bead at rest in field of energised electromagnet prior to de-energization.

FEMM also allowed us to investigate flux density within the energized core to ensure that there was sufficient core material to avoid significant saturation, see Fig. 4. Due to its axis-symmetric location within the model it is the left electromagnet’s internal flux can be treated quantitatively. The results indicate a maximum flux density in the region of 1.5T, a value that, whilst beginning to saturate in the central core region, is not considered excessive.


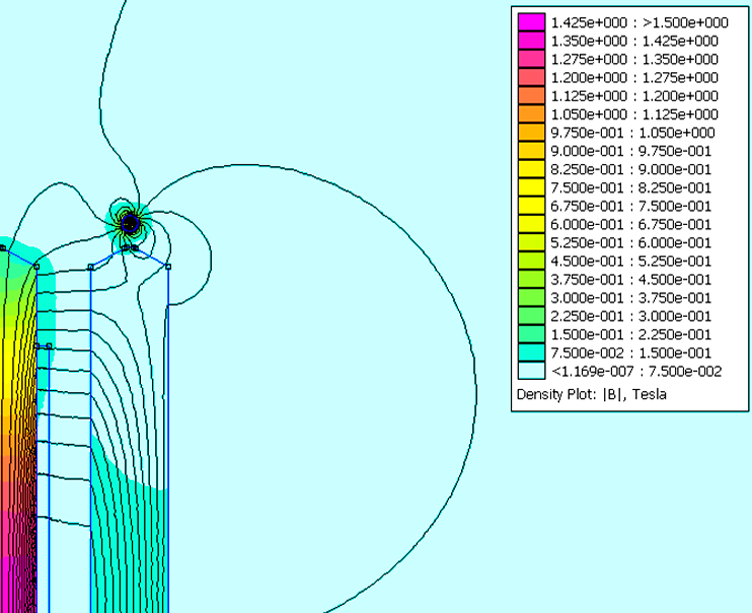


Figure 4. Core flux density of upper half of electromagnet core immediately after actuation of the next electromagnet and prior to movement of the bead. Note: contour value range has been changed from previous figures to emphasize high magnetic field B.

1. FEMM (Copyright David Meeker) is Free Public License analysis tool designed to solve static, two-dimensional magnetics problems. It provides a 'front end' to meshers and solvers and a result viewing capability. The user guide ([www.femm.info/Archives/doc/manual.pdf](http://www.femm.info/Archives/doc/manual.pdf)) provides a full discussion of the underlying governing equations, boundary conditions and solution methodologies. [↑](#footnote-ref-1)
